# Repurposing ivacaftor to attenuate LPS-induced acute lung injury: evidence from a non-cystic fibrosis model

**DOI:** 10.3389/fphar.2026.1787276

**Published:** 2026-06-01

**Authors:** Xiaoxuan Han, Yimin Zhu, Christopher W. Armstrong, Danni Li, Rachel McQuade, Andrew Jarnicki, Elena K. Schneider-Futschik

**Affiliations:** 1 Department of Biochemistry and Pharmacology, School of Biomedical Sciences, Faculty of Medicine, Dentistry and Health Sciences, The University of Melbourne, Parkville, VIC, Australia; 2 Gut- Laboratory, Department of Medicine Western Health, Melbourne University, Melbourne, VIC, Australia

**Keywords:** acute lung injury, cystic fibrosis transmembrane conductance regulator potentiator, inflammation, intratracheal administration, ivacaftor, lipopolysaccharide, lung inflammation, non-cystic fibrosis model

## Abstract

In this study, we explicitly evaluate the anti-inflammatory effects of ivacaftor as a route-of-delivery comparison (intraperitoneal vs. intratracheal) in a reproducible LPS-induced lung inflammation model. We evaluated the effects of ivacaftor [40 mg/kg, administered either intraperitoneally (IP) or intratracheally (IT)] on lipopolysaccharide (LPS, 8 μg)-induced lung inflammation in 8–10-week-old female C57BL/6 mice. Mice were euthanised at 24 or 72 h post-treatment, and serum, bronchoalveolar lavage fluid (BALF), and lung tissue were collected for analysis. Inflammation was assessed by measuring immune cell infiltration, fluorescence-activated cell sorting (FACS)-based leucocyte profiling, and histological changes. Ivacaftor concentrations in the samples were quantified using multiple reaction monitoring liquid chromatography–mass spectrometry (MRM–LCMS). At 24 h, both IT and IP ivacaftor significantly reduced the total BALF cell counts (∼40% reduction) and neutrophil infiltration compared to the LPS-only controls (*p* < 0.05). Inflammatory responses were reduced following ivacaftor treatment, as reflected by lower BALF cellularity, reduced neutrophil infiltration, and improved histological outcomes. Histological analysis showed reduced alveolar wall thickening and immune cell infiltration in ivacaftor-treated lungs. FACS confirmed lower frequencies of neutrophils and macrophages. Ivacaftor concentrations were higher in lung tissue following IT administration. Our findings indicate that ivacaftor has a potential anti-inflammatory effect, unrelated to cystic fibrosis transmembrane conductance regulator (CFTR) function, in a murine model of LPS-induced acute lung injury. These results offer insights into inflammation mechanisms and could inform future clinical treatments.

## Background

Acute lung injury (ALI) and its most severe form, acute respiratory distress syndrome, are clinical conditions characterised by sudden-onset respiratory failure, bilateral pulmonary infiltrates that are indicative of oedema, and normal cardiac filling pressures. ALI in humans is histopathologically characterised by neutrophilic alveolitis (Matute-Bello et al., 2008). Studies that have collected lung alveolar fluid—either through bronchoalveolar lavage (BAL) or direct aspiration of oedema fluid via deep airway suction—consistently show a hallmark acute neutrophilic inflammatory response (Pittet et al., 1997). This is illustrated by fibrin-rich proteinaceous exudates containing a complex mix of pro- and anti-inflammatory cytokines (Pittet et al., 1997).

Cystic fibrosis transmembrane conductance regulator (CFTR) modulator therapy was a pivotal progression in cystic fibrosis (CF) therapy development. Ivacaftor is the first clinically approved CFTR potentiator, and it functions by binding to the CFTR protein, increasing its opening time and the transport of chloride ions (Jennings and Flume, 2018). Ivacaftor is a small-molecule quinolinone-based CFTR potentiator with a highly lipophilic structure and limited aqueous solubility (Ghelani and Schneider-Futschik, 2020; Schneider et al., 2015; Schneider et al., 2016a). While these properties support membrane permeability and oral bioavailability, they also create formulation challenges compared to more water-soluble anti-inflammatory agents that are commonly delivered in simple aqueous preparations. Accordingly, solvent systems or specialised delivery approaches may be required when repurposing ivacaftor for alternative routes such as pulmonary administration.

Ivacaftor improves lung function and mucociliary clearance (Rowe et al., 2014). Ivacaftor partially corrects airway inflammation in a humanised G551D rat (Green et al., 2021). However, administration of ivacaftor led to only a partial resolution of inflammation in rats exposed to an external trigger [such as lipopolysaccharide (LPS)], indicating that CFTR activation alone may be insufficient to fully resolve lung inflammation in patients with cystic fibrosis.

Although ivacaftor is well-established in clinical use for CF, its immunomodulatory effects outside the correction of ion transport remain incompletely understood. In particular, it is unclear whether CFTR potentiation can sufficiently modulate inflammatory responses under conditions of acute inflammatory challenge.

Lung delivery offers a key advantage in acute situations such as ALI by enabling rapid and localised treatment directly at the site of inflammation or infection (Han et al., 2023). This can lead to faster therapeutic effects, reduced systemic side effects, and improved drug efficacy—particularly important when time is critical, such as in ALI or severe respiratory infections (He et al., 2022).

Therefore, in this study, we analysed the effects of lung-delivered and systemic ivacaftor administration in an acute, non-CF LPS-induced model of lung inflammation, independent of its established CFTR-related functions, to address this knowledge gap and clarify its potential role in regulating inflammation beyond its current clinical applications.

## Methods

### Materials and methods

#### Animals

Wild-type C57BL/6 mice were used to isolate the anti-inflammatory effects of ivacaftor from its CFTR-potentiating role, allowing for a clearer assessment of drug action in a controlled non-CF background. This approach also ensured a reproducible response to LPS-induced inflammation without the added complexity of CF-related pathology. Adult female C57Bl/6 mice (8–10-week-old mice, ∼20 g; Animal Resources Centre, Perth, Australia) were used for all the experiments. The mice were housed at 20 °C ± 1 °C with a 12 h light/12 h dark cycle and supplied with a balanced diet of irradiated chow and water *ad libitum*. All animal experiments were conducted in accordance with National Health and Medical Research Committee guidelines, were approved by The University of Melbourne Animal Ethics Committee (Ethics ID: 10077), and adhered to the ARRIVE guidelines (1). The number of animals was pooled after three independent experiments.

### LPS administration/LPS-induced lung inflammation

Experiments for finding the LPS dose were conducted to determine the LPS dose causing lung inflammation (Supplementary Figure S1). For all experiments, mice (weight range 17 g–24 g) were treated with *P. aeruginosa* LPS (8 µg in 50 µL of sterile saline, referred to as LPS-treated group) or 50 µL of sterile saline (referred to as the sham group) by a single IT injection.

### Drug administration

A total of 40 mg/kg of ivacaftor (Clearsynth India) was administered to the mice via IP or IT. Intraperitoneal and intratracheal administration in mice were selected to model systemic and local drug exposure, respectively, providing pharmacokinetic parallels to human oral and inhaled delivery routes that are commonly used for ivacaftor.

The ivacaftor dose of 40 mg/kg and a single administration were based on prior studies demonstrating effective tissue exposure and anti-inflammatory activity in murine models (Harwood et al., 2022; McQuade et al., 2023).

Ivacaftor for IP administration was dissolved in a mixture of DMSO/PBS (40%/60%) solution (the minimum volume required to fully dissolve ivacaftor; Chem-Supply Pty Ltd.), and the ivacaftor for IT administration was dissolved in a mixture of 87.6% DMSO, 0.6% PEG, 1.4% Tween, and 10.4% water. The formulation was selected based on prior murine studies demonstrating adequate ivacaftor solubility, tissue exposure, and biological activity, although the use of relatively high DMSO concentrations is acknowledged as a limitation (Harwood et al., 2022; McQuade et al., 2023). Experimental groups included sham controls (no LPS exposure or ivacaftor treatment), LPS-only controls, and LPS-exposed animals treated with ivacaftor via either IP or IT administration. Separate vehicle-only control groups were not included. Vehicle formulations for ivacaftor delivery were selected based on previously published methods demonstrating effective solubilization and *in vivo* administration of ivacaftor (Harwood et al., 2022; Harwood et al., 2021).

Half the cohort was collected at the 24-h mark; the remaining animals were collected at 72 h. Bronchoalveolar lavage fluid (BALF), lung, and blood samples were collected, and inflammatory marker assessments were further analysed.

### Intratracheal LPS administration

A single dose of *P. aeruginosa* LPS (8 µg in sterile saline) was administered via IT into the lungs (Supplementary Figure S1). Briefly, mice were anaesthetised with an IP injection of a ketamine/xylazine solution (90 mg/10 mg/kg). Once the appropriate level of anaesthesia was achieved, LPS was administered using a MicroSprayer Aerosolizer (Penn-Century, Wyndmoor, PA, United States). Subsequently, the animals were treated with either IT or IP ivacaftor (dosing above).

### Ivacaftor treatment groups

Mice were randomly divided into four groups, namely, a control group, an LPS group, an ivacaftor (40 mg/kg) + LPS group (8 µg in 50 µL of sterile saline) (IP), and an ivacaftor (40 mg/kg) + LPS group (8 µg in 50 µL of sterile saline) (IT). The mice of the ivacaftor (40 mg/kg) + LPS (8 µg in 50 µL of sterile saline) group received an IP injection of ivacaftor (40 mg/kg) 1 h before LPS treatment. Mice in the ivacaftor + LPS group (40 mg/kg) received IT administration using the same method as other groups to avoid procedural differences that could confound results. An equal volume of PBS, instead of ivacaftor, was administered to the LPS and control groups. Then, 1 h later, 8 µg of LPS in 50 µL of PBS was introduced intratracheally to produce lung inflammation. Control mice received 50 µL of PBS. Half of the mice were humanely sacrificed 24 h after LPS treatment, and the remaining animals were sacrificed at 72 h. Then, the bronchoalveolar lavage fluid (BALF) was collected for subsequent analysis. The primary study endpoints included BALF total cell counts, differential leucocyte counts, FACS immune profiling, histological lung injury scores, and ivacaftor concentration measurements. In contrast, endpoints such as the total BALF cell counts, histological lung injury scores, and alveolar wall thickening were used to assess general lung injury and tissue-level inflammation.

### BALF collection

After either 24 h (full inflammation) or 72 h (resolved inflammation), mice were euthanised by an overdose of IP injection of ketamine/xylazine (90/10 mg/kg) and pentobarbitone (200 mg/kg). In brief, a small incision was made in the trachea, a 19G needle was inserted into the trachea, and the airways were perfused thrice with PBS. The collected BALF was stored on ice to ensure the cell survival rate. The BALF was used to measure the immune cells and ivacaftor concentrations. The BALF was centrifuged at 4,000 rpm for 5 min to separate the cellular and acellular components. BALF was stored at −80 °C, and the cellular component was used for further analysis.

### Total cell count

Fresh BALF was briefly vortexed after collection for even cell distribution; an aliquot was mixed with 0.05% trypan blue solution to identify dead cells, and the solution was added to a haemocytometer. The haemocytometer was placed under ×40 magnification, and the number of live cells was determined.

### Differential cell count

To broadly determine the BALF cell phenotype, a cytospin was performed using a Shandon CytoSpin 3 machine to evenly distribute the cells on a glass slide. The slides were then stained with Diff-Quick^TM^ for the differentiation of cell components. Neutrophils, macrophages, and lymphocytes were differentiated based on the colour, shape, and size. The slides were then assessed under ×40 magnification. Differential cell counts were performed independently by two researchers (XH and NP) in a single-blinded manner to minimise bias. Counts from both the assessors were reviewed for consistency, and the final values used for statistical analysis were derived from the consensus/averaged differential counts.

### Serum collection

Blood was drawn into a syringe from the abdominal aorta by a 19G needle and transferred into an Eppendorf tube. The tubes were left at room temperature for 20 min to coagulate. Clotted blood was subsequently centrifuged for 5 min at 2,000 rpm to isolate and collect the serum. The serum was stored at −80 °C for further analysis.

### Lung histology and tissue fixation

An opening in the trachea was made, through which a plastic needle was inserted and secured by string to avoid fluid leakage. The diaphragm was removed before the fixation process to visualize correct lung inflation. A catheter was connected to the plastic needle, and formalin was used to inflate and fix the lungs under 20 cm of atmospheric pressure for 10 min. The trachea was ligated, and the lung was dissected out and transferred into a formalin bath for 3 h. The formalin was then exchanged with PBS for histological assessment.

The tissue was processed for histological staining by the Melbourne University histology platform using standard techniques. Slides were stained with either Alcian blue-PAS (AB-PAS) for mucin-producing cell identification in the lung tissue and haematoxylin and eosin (H&E) to identify the tissue structure and inflammatory cell localisation. The histological lung inflammation scores were evaluated according to the structural change, airway, blood vessel thickness, and cell infiltration (Supplementary Table S1). Ten random micrographs (10x) were taken per lung, and each micrograph was assessed, with the averages score used to determine the final inflammation score per lung.

### Fluorescence-activated cell sorting preparation and analysis

The BALF was centrifuged for 5 min at 1,200 RPM, and the cells were washed with PBS. Cells were resuspended in FACS buffer (PBS+0.1% NaN3), and dead cell discrimination was determined with the addition of LIVE/DEAD™ Fixable Aqua (Thermo Fisher Scientific, MA, United States). The cells were then washed and stained with antibodies, as listed in Supplementary Table S2. Cells were then washed thrice with PBS and resuspended in 4% paraformaldehyde (PFA)/PBS solution for 10 min to fix the cells. The PFA/PBS solution was removed, and the cells were resuspended in PBS for FACS analysis. Cell data were collected using a Beckman Coulter CytoFLEX S Flow Cytometer and analysed using FCS Express (*De Novo* software, Pasadena, CA, United States). A minimum of 15,000 events were collected for evaluation.

For FACS analysis, doublets and dead cells were excluded (singlet = 97.7% and live cells = 90.23%), followed by sorting on the common immune cell marker CD45 (96.41%). The cells were then differentiated into various immune cell subtypes, and the percentage of each cell type were determined (Supplementary Figures S4, S5).

### Liquid chromatography–mass spectrometry

The method details for liquid chromatography–mass spectrometry (LC-MS) analysis have been previously described (Schneider et al., 2016b; Reyes-Ortega et al., 2020; Schneider et al., 2017). Briefly, proteins were precipitated using 0.1% formic acid/acetonitrile (ACN). The sample mixtures were then centrifuged at 3,000 rpm (BALF) or 10,000 rpm (serum) for 5 min. Supernatants were collected and analysed using a Shimadzu LCMS-8050 triple quadrupole mass spectrometer. Ivacaftor and its metabolite at the M1 and M6 concentrations were measured.

### Statistical analysis

Sample sizes were calculated based on the preliminary data to achieve 80% power to detect a 30% difference in inflammatory cell counts with a significance level of 0.05; statistical analyses were performed using ANOVA, followed by Tukey’s *post hoc* test for multiple comparisons to control for type I error. Experimental results were shown as the mean ± standard error of the mean (Khan et al., 2019), unless otherwise stated. One-way ANOVA was used to determine the significance for multiple sample groups (a *p*-value of <0.05 was considered statistically significant). All graphs and statistical analysis were generated using GraphPad PRISM™ (version 9.3.1).

## Results

### Effect of ivacaftor treatment on airway immune cell numbers

**FIGURE 1:**
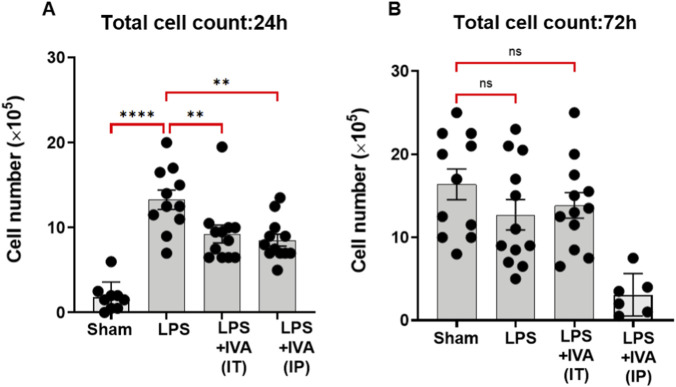
Total cell count results of all treatment groups from the 24-h **(A)** and 72-h **(B)** cohorts. The graphs demonstrate the total cell count result of LPS, ivacaftor 40 mg/kg, and sham groups (PBS only) comparisons within the 24-h and 72-h cohorts **(A,B)**. Data were collected immediately after BALF collection, and each point is an individual mouse. Data were analysed using one-way analysis of variance comparisons test and are presented as the mean ± SEM. ns = *p* > 0.05 (not significant), **p < 0.01, ***p < 0.005, and ****p < 0.0001; n = 6–12 mice/group.

The effect of ivacaftor treatment was observed to significantly reduce lung inflammation by decreasing the total number of leucocyte cells in the BALF after 24 h. This effect was evidenced by a notable decline in the total leucocyte cell count, as shown in Figure 1A. However, this beneficial anti-inflammatory impact was short-lived, as the total leucocyte cell numbers returned to baseline levels after 72 h, as depicted in Figure 1B. These findings indicate that while ivacaftor is effective in reducing acute inflammation within a short period, its effects are not sustained over a longer duration. This information emphasizes the importance of considering the timing and duration of ivacaftor treatment for optimal therapeutic outcomes in managing lung inflammation. Further research may be necessary to understand the mechanisms behind the transient nature of its anti-inflammatory effects and develop strategies for maintaining its benefits over a prolonged period.

### Effect of ivacaftor treatment on changes in body weight

As lung inflammation can lead to a reduction in body weight, we monitored changes in body weight at 24 and 72 h following LPS exposure. At 24 h, the LPS group (no drug treatment) showed the greatest weight loss, with an average change of −2.12 g ± 0.38 g (Supplementary Figure S2A). Both IT and IP ivacaftor treatments reduced LPS-induced body weight loss, with changes of −1.433 g ± 0.139 g and −1.439 g ± 0.127 g, respectively. However, no statistically significant differences were observed between the four groups (*p* = 0.103 and *p* = 0.107, respectively) (Supplementary Figure S2A). At 72 h, neither IP nor IT ivacaftor treatments significantly affected LPS-induced body weight changes, though there was a trend toward reduced weight loss.

### Effects of ivacaftor treatment on lung leukocytes

**FIGURE 2:**
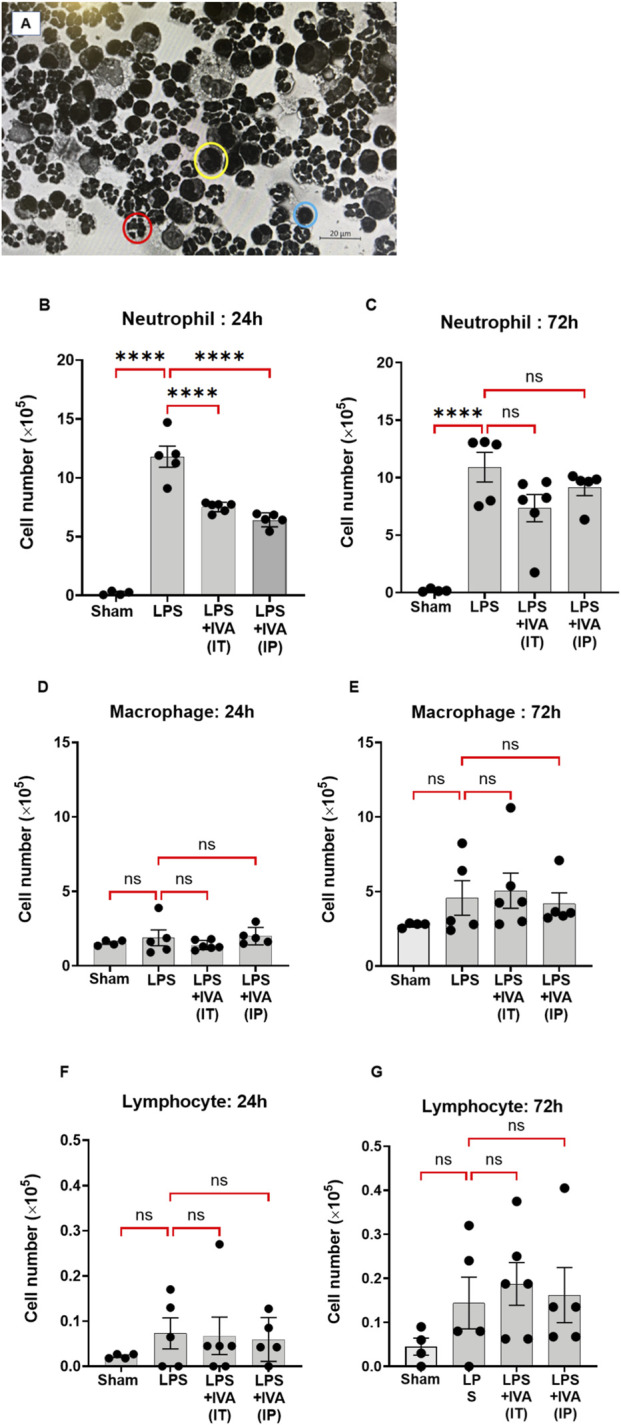
Changes in leucocyte subsets in the BALF. **(A)** Representation of a cytospin used to assess BALF leucocyte subtypes using standard morphological criteria as indicated, namely, red (neutrophil), yellow (alveolar macrophage), and blue (lymphocyte). **(B,C)** Differential cell count results of 24-h and 72-h neutrophils between treatment groups. **(D,E)** Differential cell count results of 24-h and 72-h alveolar macrophages between treatment groups. **(F,G)** Differential cell count results of 24-h and 72-h lymphocytes between treatment groups. Data were collected immediately after BALF collection, and each point is an individual mouse. Data were analysed using one-way analysis of variance comparisons test and are presented as the mean ± SEM. ns = *p* > 0.05 (not significant), ***p < 0.005, and ****p < 0.0001; n = 4–6 mice/group.

The differential white blood cell count provides a precise indicator of the severity of the inflammatory response (Figure 2A). The LPS (no drug) group showed a significant increase in neutrophil count compared to the sham-treated group (*p* < 0.0001) at both the 24-h and 72-h time-points (Figures 2B,C). This increase is indicative of a strong inflammatory reaction. On the other hand, administration of ivacaftor did not affect the numbers of macrophages or lymphocytes at either time-point (Figures 2D–G). This indicates that while ivacaftor may not influence these specific cell types, it could still play a role in other aspects of the immune response. To ensure the accuracy and reliability of these findings, differential cell counts were performed blindly by two independent researchers. These findings contribute valuable insights into the specific immune cell dynamics in response to LPS-induced inflammation and the selective role of ivacaftor in this context. Understanding these cells count variations is crucial for developing targeted therapies that can modulate the immune response without broadly affecting all the white blood cell types.

### Effects of ivacaftor treatment on white blood cells using FACS

**FIGURE 3:**
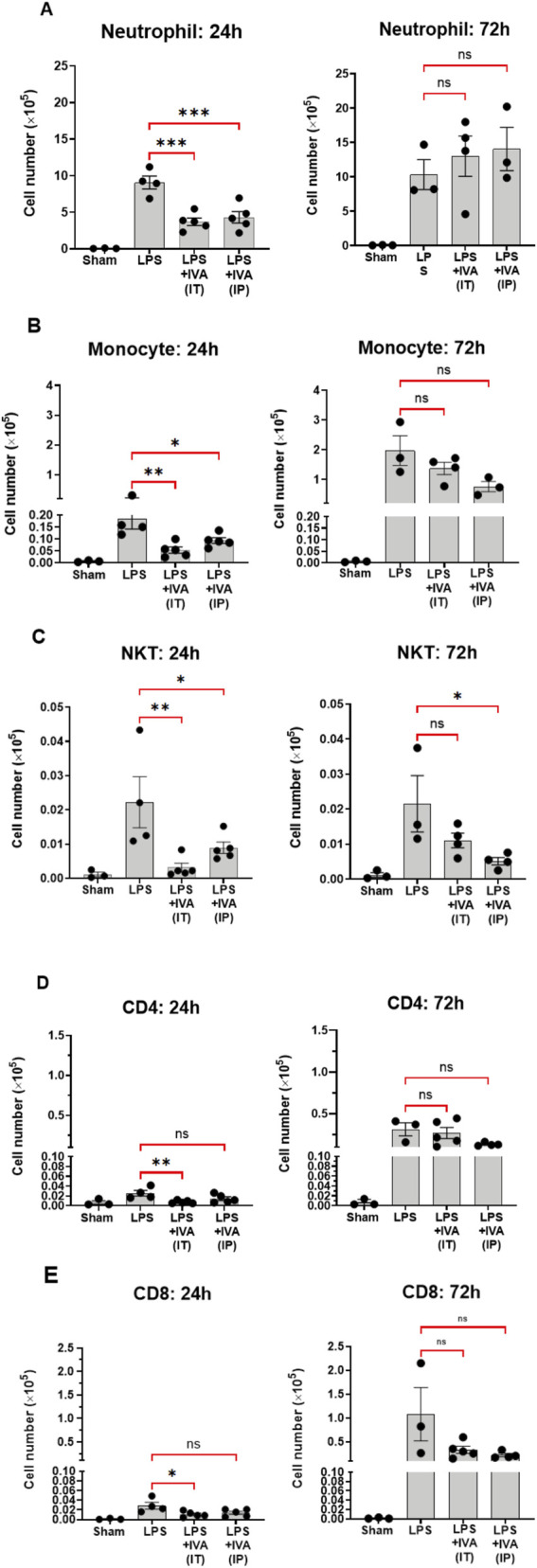
Representative FACS results. The amount of CD11b^+^ Gr1^+^ neutrophils **(A)**, CD11b^+^ CD11c^+^ monocytes **(B)**, NKT cells **(C)**, CD4 T lymphocytes **(D)**, and CD8 T lymphocytes **(E)** in the 24-h and 72-h cohort animals’ BALF were measured using FACS. Data were collected immediately after BALF collection and the staining process. Each point is an individual mouse. The data were presented as the mean ± SEM. ns = *p* > 0.05 not significant, * = *p* < 0.05, ** = *p* < 0.01, and *** = *p* < 0.005; n = 3–5 mice/group.

The BALF of all three LPS groups (no drug, ivacaftor IT, and ivacaftor IP) showed high levels of neutrophils (83.21%, 71.91%, and 84.67%, respectively) compared to that of the sham treated group, which contained mostly alveolar macrophages (79.67%) (Supplementary Figures S4,S5). Furthermore, IT ivacaftor administration significantly decreased the number of neutrophils, monocytes, NK cells, NK T-cells, B-cells, CD4 T lymphocytes, and CD8 T lymphocytes compared to that in the LPS group in the 24-h cohort (*p* = 0.0001, *p* = 0.0016, *p* = 0.0051, *p* = 0.0041, *p* = 0.0119, *p* = 0.0082, and *p* = 0.0154, respectively) (Figure 3; Supplementary Figure S6). Ivacaftor administration did not affect alveolar macrophages (Supplementary Figure S6A).

Interestingly, IP ivacaftor administration only decreased the neutrophils, monocytes, and NK T-cells at 24 h after LPS administration (*p* = 0.0004, *p* = 0.0239, and *p* = 0.0454, respectively) (Figures 3A–C). No significant difference was observed at 72 h (Figure 3).

### Histopathological changes

**FIGURE 4:**
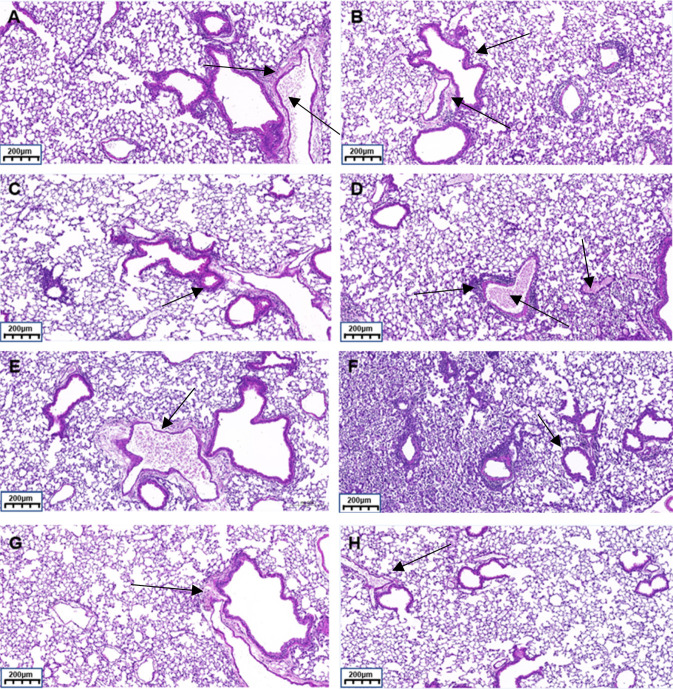
Representative images are shown; quantitative inflammatory scoring used for statistical analysis is presented in Figures 4A,B (haematoxylin and eosin staining, 10x). **(A,B)** LPS group (24 h and 72 h, respectively). **(C,D)** LPS + IVA IP group (24 h and 72 h, respectively). **(E,F)** LPS + IVA IT group (24 h and 72 h, respectively). **(G,H)** Sham groups (PBS only). Arrows/arrowheads indicate representative areas of inflammatory cell cuffing, alveolar inflammatory infiltrates, and perivascular/peribronchial inflammation used for histopathological scoring. Images are representative; group differences were determined using blinded quantitative inflammatory scoring.

**FIGURE 5:**
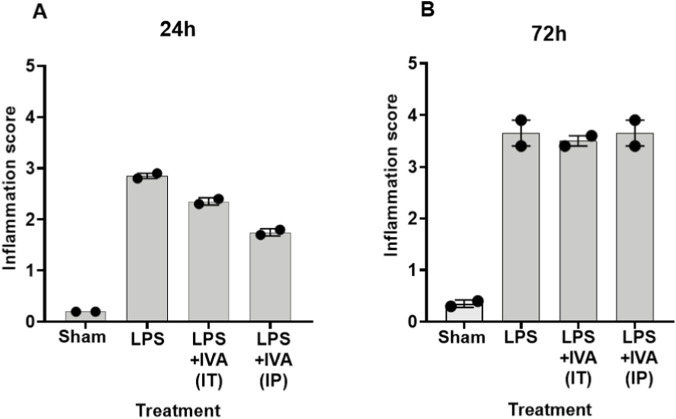
Histology inflammation score evaluation results. **(A)** Lung inflammation scores at 24 h post-treatment. (scored 0–6, as detailed in Supplementary Table S1). **(B)** Lung inflammation scores at 72 h post-treatment. Each point represents one mouse. Data are presented as the mean ± SEM. Histological scoring was performed on all available animals within each treatment group; representative images are shown separately in Figure 4.

Lung tissue was examined and assigned an inflammatory score based on inflammatory cell cuffing around the airways and blood vessels, along with alveolitis, as detailed in Supplementary Table S1. Across all the experimental groups, inflammatory scores tended to increase between 24 and 72 h post-exposure. Ivacaftor-treated groups, particularly the IT treatment group, showed lower inflammatory scores relative to the corresponding LPS control group, which is consistent with the BALF and FACS findings (Figures 4 and 5). Although inflammation remained evident at 72 h, these data indicate that the route of ivacaftor administration may influence the magnitude of the inflammatory response. Given the limited histological sample size, these findings should be interpreted alongside the complementary cellular and immunophenotyping outcomes.

### BALF and concentration quantification of serum ivacaftor and the metabolites

**FIGURE 6:**
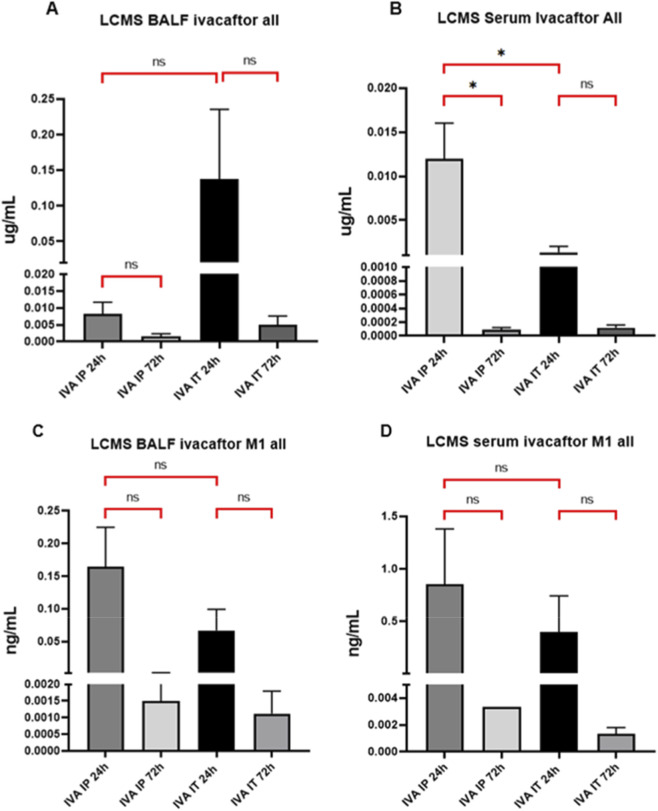
Results of ivacaftor and metabolite concentration measurements. **(A)** Ivacaftor concentration in the BALF of each treatment group. **(B)** Ivacaftor concentration in the serum of each treatment group. **(C)** Ivacaftor M1 metabolite concentration in the BALF of each treatment group. **(D)** Ivacaftor M1 metabolite concentration in the serum of each treatment group. The data are presented as the mean ± SEM.

The concentration of ivacaftor and the metabolites M1 and M6 in the BALF and serum were measured using multiple reaction monitoring liquid chromatography–mass spectrometry (MRM-LC-MS). As expected, at 24 h, ivacaftor concentrations were high in the lung after IT administration vs. IP administration (ivacaftor IT 0.137 ± 0.087 μg/mL) (Figure 6A). Unsurprisingly, concentrations in the serum were higher after IP administration vs. IT administration (ivacaftor IP 0.012 ± 0.004 μg/mL vs. ivacaftor IT 0.0013 ± 0.0007 μg/mL) (Figure 6B). Notably, the metabolites of ivacaftor, hydroxylmethyl-ivacaftor (M1 metabolite), and ivacaftor-carboxylate (M6) displayed higher concentrations in the BALF and serum at 24 vs. 72 h, indicating a fast metabolism of IP-administered ivacaftor to M1 (Figures 6C–F).

## Discussion

Numerous studies using clinical patient data or *in vitro* murine models have demonstrated that ivacaftor is effective in treating CF (Whiting et al., 2014; Kotha and Clancy, 2013; Harrison et al., 2013; Ramsey et al., 2011; Birket et al., 2020). However, using ivacaftor as an anti-inflammatory compound for ALI caused by LPS insult is a currently untapped therapeutic area. In this study, we analyse the anti-inflammatory effect of ivacaftor on LPS-induced lung inflammation. We assessed inflammatory cell extravasation into the BALF, immune cell populations, and lung histology. Our results demonstrate that ivacaftor has anti-inflammatory properties that ameliorate lung inflammation to some extent.

Inflammatory cell extravasation to the lungs as part of lung infection, reflected by the total number of cells detected in the BALF, is used as a general marker of inflammation severity. IT administration of LPS triggers a rapid innate immune response, which is characterised by an initial increase in BALF neutrophil counts, followed by a later macrophage and lymphocyte infiltration from 24 to 48 h after stimulus exposure (Asti et al., 2000). Interestingly, the 24-h ivacaftor treatment groups exhibited lower levels of lung inflammation, as measured by reduced inflammatory immune cell counts in the BALF. Neutrophils play a critical role in lung inflammation by initiating, propagating, and resolving inflammation through their migration into the lungs, where they perform various pro-inflammatory functions. While neutrophils are essential for infection clearance, excessive infiltration and prolonged presence in the lung, along with continued production of antibacterial components, can lead to tissue damage and worsen disease outcomes. Consistent with our 24-h results, neutrophils are typically the predominant immune cells to extravasate into tissues during the acute phase of inflammation. A possible explanation for the observed reduction in neutrophil infiltration may be effect of ivacaftor on improving impaired neutrophil degranulation, as described by Bratcher et al. (2016) and Pohl et al. (2014). In models of ALI, neutrophils accumulate in the lungs and exhibit heightened activation of the kinases Akt and p38, along with increased nuclear translocation of the transcription factor NF-κB (Abraham, 2003). This is accompanied by elevated production of pro-inflammatory cytokines, particularly those regulated by NF-κB (Abraham, 2003).

Additionally, independent groups show that ivacaftor administration leads to the reduction of IL-1β levels (Green et al., 2021; Harwood et al., 2021; Jarosz-Griffiths et al., 2020). IL-1β is capable of directly or indirectly inducing neutrophil production and migration (Pyrillou et al., 2020). Neutrophil accumulation has been observed in people with ALI and people with CF, as they undergo necrosis rather than apoptotic processes (Ganter et al., 2008). The necrotic pathway leads to the release of pro-inflammatory mediators, which further amplifies the neutrophil influx, likely increasing the mucus viscosity (Nichols and Chmiel, 2015). Neutrophils are present in excess numbers but fail to resolve the pulmonary insults (Ganter et al., 2008; Esther et al., 2019). It has even been suggested that neutrophils express CFTR protein on their cell surface, which highlights a potential correlation between CFTR impairment (as seen in CF and chronic obstructive pulmonary disease) and neutrophil dysregulation (Lara-Reyna et al., 2020; Painter et al., 2006; Mall et al., 2023). In the airways, neutrophil accumulation not only worsens pro-inflammatory conditions but also contributes to compromised mucociliary clearance. The decomposition of neutrophils releases large amounts of intracellular contents, including various pro-inflammatory chemoattractant (molecular agents that cause immune cell influx) such as IL-8, tumour necrosis factor-alpha, and macrophage inflammatory protein-1 alpha, which further amplify the neutrophil influx and the subsequent inflammation (Nichols and Chmiel, 2015). Furthermore, neutrophils associate with mucus accumulation and lung injury due to the release of pro-inflammatory intracellular content, which worsens the longer the inflammation exists (Khan et al., 2019; Nichols and Chmiel, 2015). The marked reduction of neutrophils that we observed at 24 h likely leads to less swelling in the lung and increases the airway space. Other studies have shown that granulocyte products expression, altered function, and surface marker expression analysis are further tools to assess the vicious cycle of inflammation (Bratcher et al., 2016; Pohl et al., 2014).

Alveolar macrophages are the first line of immune defence against pathogens, which is activated by various pathogens, such as LPS via Toll-Like Receptor 4 (TLR4) activation (Lorenz et al., 2002). Alveolar macrophages play a critical role in inflammation resolution, as it mediates bacterial killing, and the M2 phenotype macrophages secrete anti-inflammatory cytokines, including IL-4 and IL-13, to resolve inflammation (Meyer et al., 2009). Macrophages have also been shown to express CFTR on their cell surface, and previous studies have demonstrated improvements in macrophage function in ivacaftor-treated pwCF (Zhang et al., 2018; Yoshimura et al., 1991). The CFTR expression on macrophages affects cytokine production, leading to the overproduction of pro-inflammatory cytokines such as soluble CD14 and impaired phagocytosis (Simonin-Le Jeune et al., 2013). Recent studies have also emphasized the influence of CFTR on macrophage polarization as the absence of CFTR protein on macrophages leads to a poor response to IL-13 and IL-4 (Tarique et al., 2017).

The differential effects of ivacaftor on monocytes and alveolar macrophages may reflect the distinct origins and functions of these cell populations during ALI (Lorenz et al., 2002; Guha and Mackman, 2001). Circulating monocytes are rapidly recruited into inflamed lung tissue in response to LPS-driven chemotactic signalling and may subsequently differentiate into inflammatory macrophages or dendritic cells. Accordingly, the reduction in the number of monocytes observed following ivacaftor treatment may indicate the attenuation of inflammatory cell recruitment during the acute phase of injury (Guha and Mackman, 2001; Hisert et al., 2020). In contrast, alveolar macrophages are long-lived resident immune cells that maintain airway surveillance, remove debris, and promote resolution of inflammation. Preservation of alveolar macrophage numbers may, therefore, be beneficial, as ivacaftor may alter the macrophage activity or phenotype without substantially changing the resident cell abundance.

With our short-term exposure to ivacaftor IP, we additionally observed a trend of increase in macrophage activity, albeit not significantly (Supplementary Figures S4, S5, S6A). A study by Vanherle *et al.* using a post-myocardial infarction model showed that ivacaftor affects macrophage activity if used chronically (Vanherle et al., 2023). Our findings, together with the findings from the Vanherle group, emphasize the complex effect of ivacaftor on macrophage activity depending on the administration, duration, and different causes of inflammation.

Monocytes (progenitor cells of macrophages) are activated by LPS through binding to the TLR4 complex, leading to inflammation resolution by acting as phagocytes and antigen-presenting cells (Guha and Mackman, 2001; Yanez et al., 2017). Monocytes can differentiate into macrophages and dendritic cells to regulate inflammation (Yanez et al., 2017). As anticipated, our model shows high numbers of monocytes at 24 h vs. 72 hs, highlighting the states of active and resolving inflammation, respectively (Figure 3B) (Issekutz and Issekutz, 1993). We also observe that ivacaftor (both IP and IT) reduces the numbers of monocytes at 24 h significantly and shows a trend of reducing monocytes at 72 h. Research by others has shown that the triple combination of elexacaftor–tezacaftor–ivacaftor leads to the downregulation of monocyte P2X7R-induced inflammasome activation (Bratcher et al., 2016; Hisert et al., 2020; Gabillard-Lefort et al., 2022).

Furthermore, LPS directly activates NK cells via binding to TLR-4 or indirectly by releasing pro-inflammatory cytokines (IL-12 and IL-18) from activated macrophages and dendritic cells (Goodier and Londei, 2000; Varma et al., 2002; O'Connor et al., 2006). We observed a decrease in NK cells after 24 h in both ivacaftor IT and IP administration and at 72 h in the ivacaftor IP group (Figure 3C). At 72 h, ivacaftor IT administration showed a trend of reducing NK cells, which was likely due to insufficient concentrations of ivacaftor because of metabolisation. This is beneficial as decreasing numbers of NK cells can potentially lead to less IFN-γ production and, hence, improvements in lung inflammation (Green et al., 2021; Morales-Mantilla and King, 2018).

Additionally, ivacaftor IT administration was effective in reducing B cells in the 24 h group. As people with CF show higher levels of B cells in their lung compared to healthy controls, our results highlight the opportunity of using lung delivery of ivacaftor to combat lung inflammation (Azzawi et al., 1992; Hubeau et al., 2001; Yonker et al., 2015).

Overall, we observe that ivacaftor displays an acute anti-inflammatory effect by reducing inflammatory cells at 24 h. These findings are directly correlated with high drug concentrations at 24 h. As ivacaftor is metabolized by cytochrome P450s CYP3A4 to the partial hydroxymethyl-ivacaftor (M1) and the inactive ivacaftor-carboxylate (M6), clinically relevant concentrations at the respective site are essential for the acute and time-dependent anti-inflammatory effect of ivacaftor (Figure 6) (Robertson et al., 2015). We also observe that ivacaftor IT is more effective in reducing lung inflammation than ivacaftor IP. This is likely due to the i) direct application to the large absorptive surface of the lung and ii) the low enzymatic activity of the lung (Anttila et al., 1997). Hence, when combatting lung inflammation, IT administration of ivacaftor should be considered over systemic administrations as a dose increase is likely to lead to potential side effects. Local lung delivery may prove useful as a top-up administration to the clinically provided oral dose of ivacaftor.

We further acknowledge the limitations of this study. The findings herein are in wild-type mice that were studied in a hypothesis-generating foundational step toward understanding ivacaftor’s potential anti-inflammatory effects outside its known CFTR-modulating role. Our findings require further validation in CF-specific or disease-relevant models to determine translational relevance. Although inflammatory signalling molecules were not directly quantified (e.g., cytokine or NF-κB pathway analysis), inflammation was assessed using complementary approaches, including blinded histopathological scoring and FACS-based immune cell profiling. Future studies incorporating molecular cytokine analyses will be important to further define the underlying mechanisms.

Additionally, we acknowledge that IT administration is an experimental approach used primarily for precise and reproducible pulmonary delivery in preclinical settings. While IT administration allows for controlled lung exposure in mice, it is not directly equivalent to clinical inhalation delivery.

Overall, these findings provide a preclinical rationale for exploring inhaled ivacaftor as a targeted anti-inflammatory therapy in ALI and non-CF pneumonia, where localised drug delivery may offer therapeutic benefit.

## Conclusion

Our study observed that IT administration of ivacaftor, independent of CFTR function, lead to a decrease in immune cells in the lungs following LPS injury. Furthermore, we observed promising trends in decreased inflammatory cell deposits within the lung parenchyma and decreased progression of alveolitis in both ivacaftor treatment groups, indicating that further studies are required. Our findings nevertheless provide great insight and stimulate additional prospects of direct lung delivery of ivacaftor as an inhaled formulation, which may potentially ameliorate the inflammatory disbalance associated with ACL, thus alleviating disease burden and delivering better patient care and quality of life.

## Data Availability

The raw data supporting the conclusions of this article will be made available by the authors, without undue reservation.
