# Supplementary material for: Repurposing ivacaftor to attenuate LPS-induced acute lung injury: evidence from a non-cystic fibrosis model
Source: Front Pharmacol. 2026 Jun 1;17:1787276. doi: 10.3389/fphar.2026.1787276 (PMC13265339; doi:10.3389/fphar.2026.1787276)

**Repurposing Ivacaftor to Attenuate LPS-Induced Acute Lung Injury: Evidence from a Non-Cystic Fibrosis Model**

Xiaoxuan Han^1#^, Yimin Zhu^1^, Christopher W. Armstrong^1^, Danni Li^1^, Rachel McQuade^2^, Andrew Jarnicki^1^, Elena K Schneider-Futschik^1*^

**Affiliations:** ^1^Department of Biochemistry and Pharmacology, School of Biomedical Sciences, Faculty of Medicine, Dentistry and Health Sciences, The University of Melbourne, Parkville, VIC, 3010, Australia; ^2^Gut- Laboratory, Department of Medicine Western Health, Melbourne University, Melbourne, VIC, 3021, Australia;

***Correspondence:** [elena.schneider@unimelb.edu.au](mailto:elena.schneider@unimelb.edu.au)

Supplementary Figures and Tables

Figure S1.

Figure S1. Total cell count results of mild LPS vs severe LPS (dose finding experiment).

The graph shows the comparisons of total cell count results from Mild LPS group(4μg/mouse) and Severe LPS groups (8μg/mouse) of 24-hour and 72-hour cohorts. Data were collected immediately after BALF collection. Each point is an individual mouse. Data are presented as mean ± SEM. Due to n=2 mice/group.

Figure S2.

Figure S2. Effects of LPS and ivacaftor on body weight change.

Figure S2. Effects of LPS and ivacaftor on body weight change. Data show body weight changes in wild-type mice following LPS exposure (8 μg/mouse) and treatment with ivacaftor (40 mg/kg) administered via either the IT or IP route at (A) 24 hours and (B) 72 hours post-treatment. Each point represents an individual mouse. Data were analysed using one-way ANOVA with multiple comparisons and are presented as mean ± SEM. ns=P>0.05 (not significant), *P<0.05, ****P<0.0001, n=6–12 mice/group.

Figure S3.

Figure S3. Total cell count results of all groups.

The graph demonstrates the total cell count of LPS 24-hour and 72-hour, LPS + Ivacaftor IT 24-hour and 72-hour, LPS + Ivacaftor IP 24-hour and 72-hour and untreated groups. Data were collected immediately after BALF collection. Data are presented as as mean ± SEM.

Figure S4.

Figure S4. Pie chart demostration of FACS result of 24-hour LPS and ivacaftor groups

The data shows the proportion of each type of immune cells from the 24-hour cohort BALF that were analyzed by FACS. In the LPS treated groups neutrophils represent the majority of the immune cells found at 24h compared to the LPS-free group were the highest numbers were macrophages.n=3-5/group.

Figure S5.

Figure S5. Pie chart demostration of FACS result of 72-hour LPS and ivacaftor groups.

The data shows the proportion of each type of immune cells from the 72-hour cohort BALF that were analyzed by FACS. In the LPS treated groups neutrophils represent the majority, albeit in lower percentages than in the 24h group. In the LPS-free group macrophages dominated the cell numbers found. n=3-5/group.

Figure S6.

Figure S6. Representative FACS results.

The amount of Siglec F^+^ CD11c^+^ alveolar macrophages (A), NK cells (B), and B cells (C), in 24-hour and 72-hour cohort animals’ BALF were measured using FACS. Data were analysed using one-way analysis of variance comparisons test with multiple comparisons and are presented as mean ± SEM.

ns=P>0.05 not significant, *=P<0.05, **=P<0.01, ***=P<0.005, n = 3-5 mice/group.

Table S1. Histology inflammation score evaluation system.


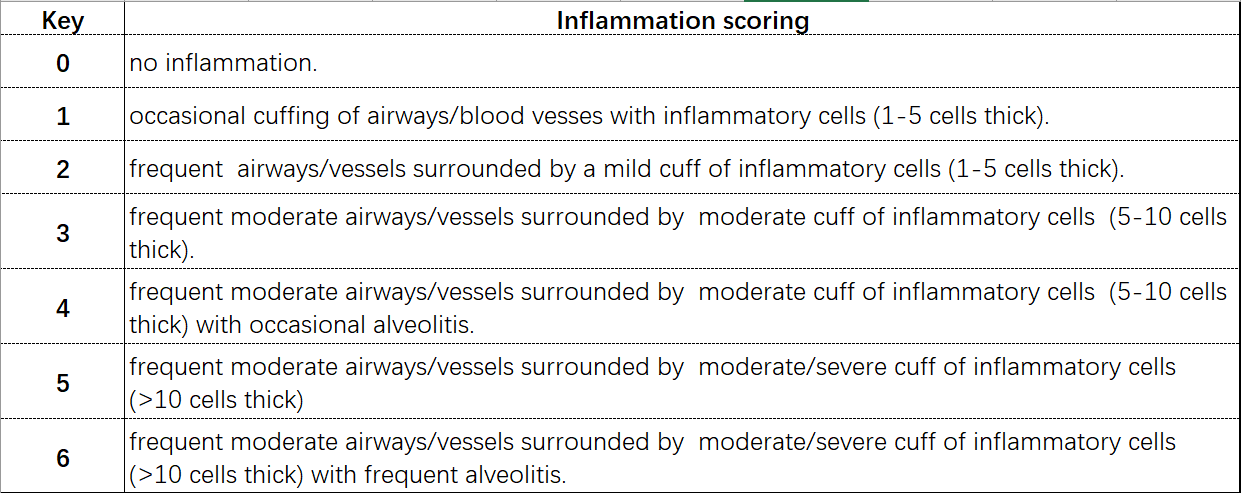


Table S2. Antibodies and corresponding fluorophores.


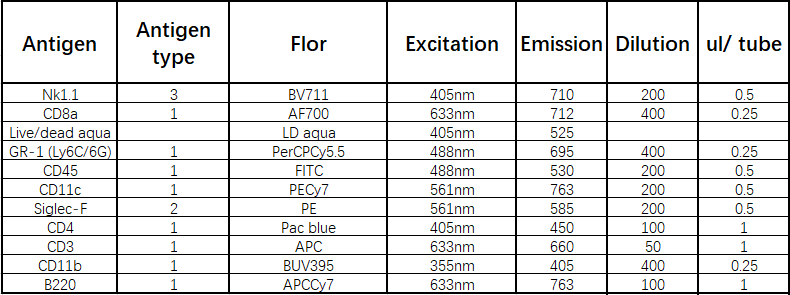

Supplement: Supplementary file 1 [file Supplementaryfile1.docx]
